# Metabolic Control in Children and Adolescents with Insulin-Dependent Diabetes Mellitus at

**DOI:** 10.4274/jcrpe.415

**Published:** 2011-12-06

**Authors:** Abdulmoein Al-Agha, Ali Ocheltree, Amr Hakeem

**Affiliations:** 1 King Abdul-Aziz University Hospital

**Keywords:** Metabolic control, insulin-dependent diabetes mellitus, HbA1c, children, adolescents

## Abstract

**Objective:** Preventing long-term diabetic complications requires good metabolic control, especially in type 1 diabetes mellitus (T1DM). We describe the metabolic control of T1DM and the factors affecting it among children and adolescents attending the Pediatric Clinic at King Abdul-Aziz University Hospital.

**Methods:** A retrospective cross-sectional study was conducted on T1DM children and adolescents who had attended the Pediatric Clinic at King Abdul-Aziz University Hospital from 2006 to 2010. Both clinical and laboratory data were reviewed for the enrolled cases. The mean age of the patients was 12.5±4.1 years. Ages ranged from 1 to 18 years (n=484: male=213, female= 271). 38.6% of the patients were pre-pubertal and 61.4% - pubertal. The patients were categorized into 3 age groups as 1-6 years (10.3%), 7-12 years (33.5%) and 13-18 years (56.2%).

**Results:**  The overall mean HbA1c was 9.4±2.4% and the duration of patient follow-up was 26±17 months. 10.3% of the patients were on conventional insulin regimens and 89.7% - on intensive insulin therapy. 31.4% had satisfactory HbA1c according to the American Diabetes Association guidelines. The duration of T1DM was 2.9±1.4 years.  The patients with diabetes duration ≤2 years (45%) had a mean HbA1c of 8.7±1.8% and those with diabetes duration >2 years (55%) had a mean HbA1c value of  9.8±2.3% (p< 0.001).

**Conclusions:** The metabolic control of T1DM children in our cohort was less satisfactory than in other studies. We recommend the promotion of physical exercise and family educational programs to improve the metabolic control of T1DM pediatric patients in our population.

**Conflict of interest:**None declared.

## INTRODUCTION

Type 1 diabetes mellitus (T1DM) is a common chronic disease in childhood. Fifty percent of subjects with T1DM are diagnosed within the first 15 years of life (1). The Diabetes Control and Complications Trial and the Epidemiology of Diabetes Interventions and Complications study demonstrated that improving metabolic control in children and adolescents with T1DM reduced the risk of diabetic complications (2,3). Previous studies have attributed poor metabolic control among adolescents to their changing physiology (pubertal growth and development) as well as to behavioral and adherence issues (4,5,6). Intensified insulin therapy resulted in a better metabolic control and reduced diabetic complications (7). Good metabolic control is crucial for the prevention of long-term diabetic complications (8). This can be achieved by daily self-monitoring of blood glucose (SMBG), multiple daily insulin injections, regular HbA1c measurements, and attention to physical activity. Exercise is an essential component in blood glucose regulation for T1DM patients, along with insulin management (9). The American Diabetes Association (ADA) emphasized the importance of ongoing education in prevention of and screening for diabetes complications (10). Diabetes education programs have small to medium beneficial effects on metabolic control (11,12) and somewhat greater effect on psychological outcomes (13). We describe the current level of metabolic control in children and adolescents attending the pediatric endocrine clinic at King Abdul-Aziz University (KAAU) Hospital and the impact of different factors such as age, pubertal stage, gender, duration of diabetes, insulin regimen, family diabetes education, and physical exercise on the metabolic control of T1DM pediatric patients in our population.

**Methodology**

**Study Design and Subjects**

This retrospective cross-sectional study was conducted on all children and adolescents who had attended the pediatric endocrine clinic at KAAU Hospital from 2006 to 2010. Inclusion criteria were: follow-up period in the pediatric endocrine clinic for more than 3 months, patient age between 1 and 18 years, and an HbA1c value >6.5%. A total of 547 patients were retrospectively reviewed and 63 were excluded from the study due to age (becoming ≥18 years during the study period) or to a follow-up period of less than 3 months. Thus, the study population consisted of 484 children and adolescents with T1DM, aged from 1 to 18 years. Mean, standard deviation (SD) and median for age values were 12.5±4.1 and 13 years, respectively. The mean (±SD) follow-up period was 26±17 months (range: 8-48 months). A total of 72 (15%) patients were followed up for 4 years. Of the study population, 213 were males (44%) and 271 were females (56%); 187 were pre-pubertal (38.6%) and 297 were pubertal (61.4%).

Duration of T1DM, attendance to diabetes education sessions and seminars, insulin regimen, rate of SMBG, Tanner staging, regularity of physical exercise, and serum HbA1c were reviewed from patient clinical and laboratory records. We categorized all patients into three age groups: toddlers and pre-school children (1-6 years), 50/484 (10.3%); school children (7-12 years), 162/484 (33.5%); and adolescents and young adults (13-18 years), 272/484 (56.2%). Prior to data entry into the study database for analysis, all collected variables were reviewed by a pediatric endocrinology consultant at KAAU for data quality assurance. In the present study, we followed the ADA guidelines for target HbA1c levels per age group. The ADA recommends a target HbA1c of approximately 8.5% in toddlers, between 7.5% and 8.5% in pre-school children, ≤8% in school children, and ≤7.5% in adolescents and young adults (10). In contrast, the more stringent guidelines of the International Society for Pediatric and Adolescent Diabetes recommend that a target HbA1c level of <7.5% should be achieved without succumbing into episodes of severe hypoglycemia (14). 

**Insulin Regimens and SMGB**

The protocol used in the pediatric endocrine clinic at KAAU regarding insulin regimens for T1DM children and adolescents is conventional insulin regimen for both toddlers and pre-school children and intensive insulin regimen for children >6 years and all adolescents. Conventional insulin regimen was defined as the administration of 2 injections of insulin/day as a combination of regular short-acting and intermediate-acting insulin before breakfast and dinner, coupled with SMBG and adjustments of insulin dosage in response to the individual's metabolic control. Importantly, hyperglycemia (depending on age) was corrected with short- or rapid-acting insulin. Intensive insulin regimen was defined as either receiving 3 insulin injections/day, and in addition receiving a basal bolus of insulin, or being on an insulin pump. Insulin basal bolus was defined as a rapid- 

or short-acting insulin injection before each meal and either a single long-acting basal dose or two intermediate-acting doses to cover the basal need for insulin between meals and during the night. Patient rate of SMBG was reviewed from the clinical records. Values of HbA1c were based on measurement at regular intervals (3 months) and then averaged to create a 4-year mean exposure variable for each enrolled subject. At KAAU, HbA1c is measured by the SEIMENS Dimension clinical chemistry system using a GLU Flex reagent cartridge. The laboratory test is the hexokinase method. 

**Diabetes Education and Physical Exercise**

Patient compliance to physical exercise and diabetes education sessions, as well as the weekly frequency of physical exercise, was assessed from a review of the hospital records of the enrolled subjects.  The patients were divided into 3 categories with respect to physical exercise: the first group did not indulge in physical exercise; the second group performed physical exercise 1-2 times/week; and the third group performed physical exercise 3-4 times/week. Compliance to physical exercise was defined as continuous physical exercise lasting ≥30 minutes/day both indoors and outdoors. Compliance to diabetes education events was defined as attendance of ≥1 session, symposium, seminar, lecture, and clinical appointment/month.

**Statistical Analysis**

The data were compiled from KAAU Hospital phoenix database. SPSS version 16.0 software was used for the analysis. Continuous variables were represented as mean (±SD) and categorical variables as percentages. Student t-test, Mann-Whitney U test, and Kruskal-Wallis test were used for comparative evaluation. When appropriate, Chi-square test and cross tabulations were applied for the analysis of categorical data. Spearman’s rank correlation coefficient was used to study the correlation between HbA1c and age. A p-value of <0.05 was taken as statistically significant for individual variables. As this was primarily a study of the entire population of T1DM pediatric patients who had attended the pediatric endocrine clinic at KAAU Hospital, sample size calculations were not performed a priori. This study was approved by the biomedical ethics department at KAAU, Faculty of Medicine. 

## RESULTS

Metabolic control became more challenging as pediatric patients advanced in age. Gender did not influence the metabolic control in our cohort (Table 1). The overall HbA1c was 9.4±2.4 and 8.8% (mean±SD and median, respectively). Metabolic control was less satisfactory in the pubertal group compared to the pre-pubertal group (HbA1c 10±2.6 vs. 8.5±1.7%, p<0.001). Of a total of 484 patients, 152 (31.4%) had satisfactory HbA1c values according to the ADA guidelines. In our cohort, 50/484 were on conventional insulin regimens (10.3%) and 434/484 were on intensive insulin therapy (89.7%); only 18/434 (4.1%) of those on intensive insulin therapy had an insulin pump. Metabolic control among the toddlers and pre-school age group was more satisfactory when compared to the school and adolescent age groups (Table 2). A positive correlation was found between HbA1c and age (r=+0.3, p<0.001). In our cohort, 138/484 patients (28.51%) were compliant to diabetes education programs. HbA1c levels were 8.2±0.5 for those who were compliant to diabetes education programs and 10±1.9 for those who were not compliant. The difference between these two groups was significant (p<0.05). Of a total of 484, 187 (38.6%) patients were compliant with SMBG ≥4 times/day; 119 of the 187 (63.6%) subjects were also compliant to the diabetes education exercises. The mean duration of T1DM was 2.9±1.4 years. In 218 of the 484 patients (45%), diabetes duration was ≤2 years and the mean HbA1c value was 8.7±1.8% In 266 (55%), diabetes duration was >2 years (mean HbA1c 9.8 ±2.3%). The difference between HbA1c values was significant (p<0.001). The duration of T1DM was 2.5±1.1 years in those with controlled HbA1c (according to the ADA guidelines) and 3±1.2 years in those with uncontrolled HbA1c. The difference was significant (p<0.05). According to the clinical records, only 35 of the 484 enrolled children (7.2%) performed regular physical exercise 3 to 4 times a week, 124 (25.6%) exercised 1 to 2 times a week, and the majority 325 (67.2%) did not perform any physical exercise on a regular basis. The mean HbA1c levels in these groups were 7.1±0.6, 8.2±0.7, and 10.3±2.7, respectively, with a p value of <0.001 (Kruskal-Wallis test).

**Table 1:**
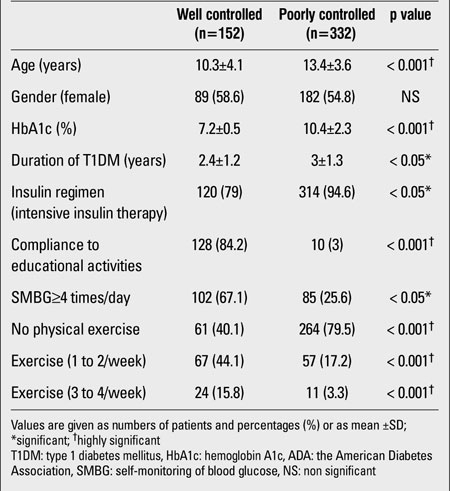
Comparison of well and poorly controlled T1DM children and adolescents according to the ADA guidelines

**Table 2:**
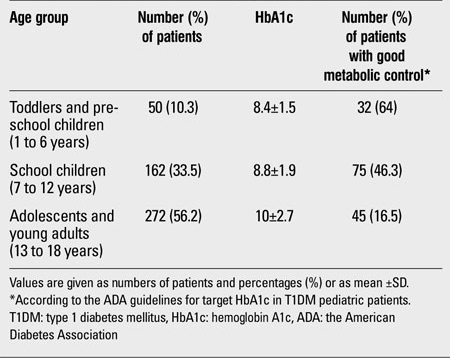
HbA1c control among the 3 age groups of the present study

## DISCUSSION

Several studies have reported on the metabolic control of T1DM children and adolescents (15,16,17,18), but this is the first to examine the metabolic control among T1DM pediatric patients in Saudi Arabia and has relevance to the planning and improvement of care in developed and less well-developed settings serving children and adolescents with T1DM. Specifically, this study demonstrates that attention to both physical exercise and diabetes education programs can be effectively established and that they are associated with improved clinical outcomes. Furthermore, despite the fact that several confounding factors play a role in metabolic control, this study shows that conventional insulin therapy may still be an appropriate therapeutic option in managing young children (≤6 years) with T1DM.  

Good metabolic control in T1DM pediatric patients reduces the incidence of complications and delays the progression of existing complications (19). It is also essential for normal growth and development (20). In our cohort, insulin regimens were not the only factor influencing metabolic control of T1DM pediatric patients. Lack of physical exercise, negligence to adhere to daily SMBG and to diabetes education programs were other contributing factors. Prevalence of T1DM among children and adolescents in Saudi Arabia was found to be 109.5/100 000 and this country has the lowest reported prevalence rate compared to  some other countries in children <6 years of age (21). This might explain why few patients in our cohort were younger than 6 years of age.

Intensive insulin therapy is the method of choice in managing T1DM pediatric patients (7,22). Paradoxically, there was a higher percentage of subjects receiving intensive insulin therapy in the poor control group. The reason for this is that the majority of our cohort (89.7%) was on intensive insulin therapy, which precludes precise comparisons between conventionally and intensively treated patients. Furthermore, the KAAU endocrine pediatric clinic prefers to start children on intensive insulin therapy as early as possible (around the age of 7 years) in order to improve their metabolic control and reduce the risk of diabetic complications. Initiation of such therapy in young children (≤6 years) is limited by problems related to non-acceptance of multiple injections due to fear of needles, fear of both the child and his/her parents of severe hypoglycemic attacks, especially in those who have had a past history of seizures during their sleep, unpredictability of a toddler's dietary intake and activity level, difficulty of adherence to daily SMBG and regular measurements of HbA1c, and lack of regular exercise. Subcutaneous insulin infusion therapy may provide yet another solution for some patients, but without government subsidy, this type of treatment is not affordable for most families (23). Indeed, very few families in our cohort (4.1%) could afford insulin pump therapy. 

Several studies reported that metabolic control was worse in adolescents compared to younger children (16,24). In our cohort, younger children had a better control compared to adolescents. Interestingly, T1DM patients ≤6 years of age who were on conventional insulin therapy had good metabolic control. Intensive insulin therapy is the preferred therapeutic approach in managing children and adolescents with T1DM (7,22). However, conventional insulin regimens consisting of two insulin injections daily may still be an appropriate therapeutic option for children ≤6 years of age, particularly for families who cannot afford intensive insulin therapy. This choice has to be made together with the patient and his/her parents upon consideration of several factors such as: age; acceptance of a restricted versus flexible diet; acceptance of a fixed versus flexible but multiple insulin injections between snacks and meals; the cultural and intellectual background of the subject and of the family; compliance; and a history of a partial remission phase. Both Tonella et al (16) and Dorchy et al (17) reported that no significant difference was observed between HbA1c levels of conventionally versus intensively treated patients. Nonetheless, it has been shown that intensive insulin treatment may result in a better metabolic control and cause less complications when compared with conventional approaches (25,26). However, compliance to intensive regimens has been shown to be weaker than compliance to conventional regimens, suggesting a mismatch between the treatment regimen proposed by the clinician and the extent to which patients and their families can manage diabetes (27).

Several studies conducted on children and adolescents with T1DM have demonstrated that both patient and family education were associated with a reduction in number of hospitalizations, emergency room visits, and a reduction in overall healthcare expenses (28,29). Diabetes education is not a one-time event that occurs at diagnosis. Indeed, many studies have shown that to be effective, educational interventions need to be both continuous and regular. Only then can these efforts lead to improved HbA1c values and decreased hospitalization rates (15,27,28,29,30). The ADA recommends that T1DM children and adolescents, especially those who are highly active, should monitor their blood glucose levels ≥4 times/day, (10). In the present study, families compliant to diabetes education events appreciated the value of SMBG and were able to manage their children's T1DM with greater success. Specialized pediatric dietitians can give more appealing advice to children and this will ultimately improve their overall adherence to a T1DM-suitable lifestyle (15). 

The ADA, Center for Disease Control and the American College of Sports Medicine all recommend that children and adolescents with T1DM should have a minimum of 30 to 60 minutes of exercise/day (10). In our cohort also, patients with T1DM who exercised regularly, enjoyed a better metabolic control. Michaliszyn et al. have reported that greater fitness levels predicted better metabolic control in adolescents with T1DM (18). Benefits of physical exercise in T1DM children and adolescents include satisfactory metabolic control, greater sense of well-being, weight control, improved physical fitness, improved cardiovascular fitness with lower pulse, lower blood pressure, and improved lipid profile (10,31). Herbst et al demonstrated that the frequency of regular physical activity was associated with lower HbA1c levels, but interestingly, without increasing the risk of severe hypoglycemia (32). Nonetheless, 10-20% of hypoglycemic episodes are associated with exercise, thus, frequent blood glucose monitoring is imperative (10).

The duration of T1DM and also the age of the patient affected the metabolic control of children and adolescents with T1DM in our cohort. Those with a <2 years duration of diabetes had lower HbA1c levels compared to those who had longer diabetes duration. Flück et al (24) reported that metabolic control was worse in pubertal adolescents compared to pre-pubertal children and also reported that patients with a duration of diabetes <2 years had better metabolic control than those with a longer duration. In our cohort, gender did not affect the metabolic control of children and adolescents with T1DM, in contrast to findings reported in several studies (17,24). Among those on intensive insulin therapy in our cohort, good metabolic control was achieved in 46.3% of the patients who were younger than 13 years, while only 16.5% of those who were older than 13 years had a good metabolic control. 

Our study had some limitations. Firstly, the unequal distribution of patients among age groups in our population precludes precise comparisons. Secondly, in this retrospective study, we were not able to assess either the rates of hypoglycemia, a parameter which is important particularly for T1DM patients on intensive insulin therapy, or the nutritional/dietary status and life style of the patients, except for their practice of physical exercise. 

From the results of this study, we conclude that the best therapeutic approach in pediatric patients with T1DM is intensive insulin therapy. However, other factors such as duration of diabetes, attendance to both diabetic education programs and physical exercise sessions also affect metabolic control in children and adolescents with T1DM. Emphasis must be made on the importance of diabetes education in helping families improve their children's metabolic control.
